# Avian Orexin: Feed Intake Regulator or Something Else?

**DOI:** 10.3390/vetsci9030112

**Published:** 2022-03-03

**Authors:** Alison Ramser, Sami Dridi

**Affiliations:** 1 Center of Excellence for Poultry Science, University of Arkansas, Fayetteville, AR 72701, USA; atramser@uark.edu; 2 Cell and Molecular Biology Program, Department of Poultry Science, University of Arkansas, Fayetteville, AR 72701, USA

**Keywords:** orexin, central regulation, peripheral regulation, metabolism, sleep-wake

## Abstract

Originally named for its expression in the posterior hypothalamus in rats and after the Greek word for “appetite”, hypocretin, or orexin, as it is known today, gained notoriety as a neuropeptide regulating feeding behavior, energy homeostasis, and sleep. Orexin has been proven to be involved in both central and peripheral control of neuroendocrine functions, energy balance, and metabolism. Since its discovery, its ability to increase appetite as well as regulate feeding behavior has been widely explored in mammalian food production animals such as cattle, pigs, and sheep. It is also linked to neurological disorders, leading to its intensive investigation in humans regarding narcolepsy, depression, and Alzheimer’s disease. However, in non-mammalian species, research is limited. In the case of avian species, orexin has been shown to have no central effect on feed-intake, however it was found to be involved in muscle energy metabolism and hepatic lipogenesis. This review provides current knowledge and summarizes orexin’s physiological roles in livestock and pinpoints the present lacuna to facilitate further investigations.

## 1. Introduction

The orexin system has gained traction in human health and disease research for its pleiotropic function and influence on the central nervous system. Narcolepsy, depression, addiction, obesity, and type 2 diabetes are just some of the human disorders and diseases now linked to the orexin system [1,2,3]. Orexins are orexigenic hypothalamic neuropeptides that function as ligands for G-protein coupled receptors responsible for numerous cell-signaling pathways and functions within the nerves and peripheral organs [4]. Their ubiquitous and abundant expression in tissues throughout the body has resulted in research covering numerous topics, such as energy homeostasis and metabolism at both the central and peripheral level, in addition to central regulation of behavior and the wake-sleep cycle.

In farm animals, research is more limited, especially in non-mammalian species. Most research has been conducted in model species such as rats and mice, but pigs, sheep, cats, and dogs have been investigated to a lesser extent [5,6,7,8]. While some characteristics of the orexin system remain the same between humans and animals, differences in how the system is regulated and its downstream effects have been found. Orexin research in animals centered first around its effect on feeding behavior and its relationship to the hypothalamic-pituitary-adrenal axis before venturing into peripheral effects and regulation [9]. These studies show that orexins have diverse mechanisms of function and effects across species, requiring further research in livestock and companion animals as well as model organisms to fully capture their modes of action and potential utilization in animal husbandry, breeding, nutrition, and medicine. This is especially true for the avian orexin system, which has been only touched upon in recent years and already shows major differences compared to orexin in humans and mammals.

In this review, the orexin system will be described, including the orexin precursor, orexin receptors, and both types of orexins—orexin-A and orexin-B. Current research on the central regulatory role of orexins will be discussed along with the known molecular signaling pathways involved. Research investigating the peripheral role of orexins in mammals and avian species will also be described. The signaling pathways and their stimuli will also be considered. Finally, emphasis on current gaps in avian orexin that merit further investigations will be highlighted.

## 2. Overview of Orexin

Genomic research during the 1990s resulted in the characterization of numerous, previously unidentified, genes with potential biological significance. Notably, several “orphan” G-protein coupled receptors were found and shown to be putative, but without known ligands [10]. Given that G-protein coupled receptors are the most targeted molecules for drugs used in clinics, the investigation into the ligands for these orphan receptors was undertaken and revealed two peptide ligands termed “orexins”, also known as hypocretin [10,11]. It has been shown that the administration of orexins into the central nervous system (CNS) resulted in increased food intake in mice and rats, and that their production was dependent on the nutritional state. Additionally, a group of neurons located in the lateral hypothalamic area of the brain produce the neuropeptide called orexin in two forms, orexin A and orexin B [10,11]. These forms are synthesized from proteolytic cleavage of the precursor, prepro-orexin. Orexin-A is composed of 33 amino acids with an N-terminal pyroglutamyl residue and a C-terminal amidation [12,13]. Its structure consists of four Cysteine residues forming sets of intra-chain disulfide bonds. This structure is conserved across mammalian species, including rats, mice, cows, sheep, pigs, and dogs [13]. Orexin-B is made up of 28 amino acid residues, and while its C-terminal is similar to orexin-A, its N-terminal is more variable [10]. Additionally, there are more differences in orexin-B amino acid sequences across mammalian species and chicken [14].

There are two known orexin receptors, orexin-1 receptor (OX_1_R) and orexin-2 receptor (OX_2_R). These are G-protein coupled receptors with OX_1_R being structurally similar to neuropeptide receptors such as the Y2 neuropeptide Y (NPY) receptor and thyrotropin-releasing hormone (TRH) receptor [10,15,16]. Both receptor genes are highly conserved across species [17]. Orexin-A has a higher affinity for OX_1_R than orexin-B, while OX_2_R is a nonselective receptor for both orexin-A and orexin-B [10,12]. This difference in affinity for OX_1_R is attributed to the N-terminal of orexin-A, which is specific and hydrophilic [18]. Within the CNS, OX_1_R is the most abundant in the locus coeruleus, but is also found in the prefrontal and infralimbic cortex, hippocampus, amygdala, periventricular nucleus, anterior hypothalamus, dorsal raphe nucleus, and laterodorsal tegmental nucleus [4,19]. OX_2_R is expressed in the amygdala periventricular nucleus, dorsal raphe nucleus, and laterodorsal tegmental nucleus, as well as in the tuberomammillary nucleus. These regions are critical for energy homeostasis responses and arousal, which will be discussed in more detail in a later section. Furthermore, the mRNA of prepro-orexin is expressed in the lateral hypothalamus area, known as a feeding center [4,9]. The orexin system is also expressed in peripheral tissues, including the kidney, adrenal gland, thyroid, testes, ovaries, jejunum, lung, pituitary gland, brown and white adipose tissues, and muscle [6,7,14,20,21,22,23,24].

## 3. Central Role of Orexin

First discovered due to its relationship with the central nervous system, research into orexin within the regions of the brain has revealed diverse behavioral and energy sensing roles.

### 3.1. Feeding Behavior, Wake-Sleep Cycle, and Stress

**Figure 1:**
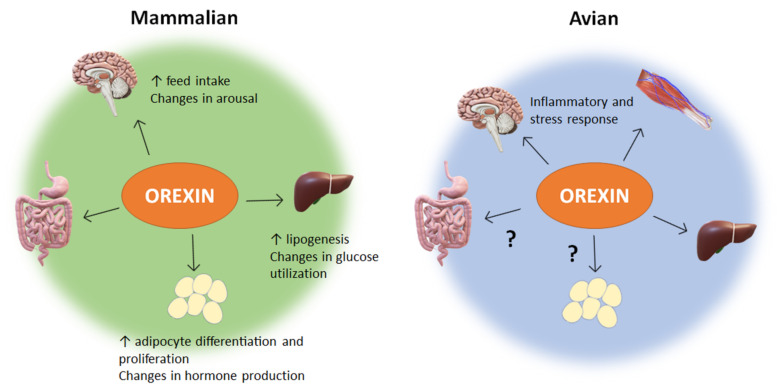
Impacts of orexin within mammalian and avian species. ? means the physiological function of orexin is still unknown or not well defined. In mammalian species of veterinary interest, such as mice, rats, sheep, cats, and dogs, orexin has been proven to be more than a neuropeptide. In addition to its central regulation of feeding behavior and sleep-wake cycles, orexin affects intestinal motility, pancreatic secretions, adipogenic factors, and energy homeostasis. In non-mammalian species such as chicken, research is limited, but shows a role of orexin in the central response to inflammatory and stress stimuli. Additionally, orexin in avian species has a peripheral influence on muscle mitochondrial dynamics and function, as well as hepatic stress response and lipogenic factors. Major gaps exist in our understanding of orexin’s influence on other peripheral tissue in avian species. Taken together, these findings demonstrate a necessity for further research into the diverse and undiscovered peripheral roles orexin plays in animal behavior, metabolism, and stress, particularly in avian species.

The lateral hypothalamic area, where orexin neurons are localized, is involved in the control of food intake and energy homeostasis (Figure 1). Indeed, intracerebroventricular (i.c.v.) injection of orexin in rats and mice induced food intake [25,26]. Orexin expression was also found in the hypothalamus of developing pigs and was shown to regulate feeding behavior in fish [27,28]. Orexin is also localized in the lateral hypothalamic area in sheep with i.c.v. injection resulting in a doubling of cumulative feed intake within 4 h compared to 24 h of intramuscular injection [29]. Orexin neurons were also shown to be sensitive to glucose levels, with increased glucose concentrations inhibiting orexin neuron firing and impacting feeding behavior and arousal in mice [30,31]. Most notably, orexin neurons in the arcuate nucleus (ARC) of the hypothalamus are glucose-inhibited and metabolism independent as the glucose response was unaffected by the glucokinase inhibitors [32]. These results further demonstrate the multifaceted regulation of the central control orexins have on feeding behavior.

Orexin in the central nervous system has also been shown to have protective effects in numerous physiological states and stressors. Studies using rat cortical neurons revealed that orexin-A and orexin-B increased neuronal viability under chemical hypoxia and protected the cell against oxidative stress [33]. These results were also seen using a hydrogen peroxide-challenged rat hypothalamic cell model, where both lipid peroxidation and apoptosis were reduced by orexin-A [34]. These data indicate that orexin supports orexin-responsive neuron survival and therefore could be regulating feeding behavior responses via neuron survival. Investigations into the molecular basis for cell survival has shown that regulation of the nuclear factor-kappa B (NF-kB) and phosphorylation of mitogen-activated protein kinase (MAPK)/P38/extracellular-signal-regulated kinase (ERK) pathways aids in attenuating inflammatory responses within the central nervous system [35]. These pathways have been implicated in lipopolysaccharide-induced neuronal stem cells and cerebral ischemia reperfusion injury. In the case of cerebral ischemia, OX_1_R mediated the MAPK/ERK/mechanistic target of the rapamycin (mTOR) pathway to inhibit excessive autophagy via orexin-A, leading to increased cellular viability [36]. Other research into cerebral ischemia revealed an inhibitory effect of orexin-A treatment on endoplasmic reticulum stress-mediated apoptosis via decreased levels of glucose related protein 78 (GRP78), phosphorylated ERK, and other related factors that were induced under injury [37]. This study provides a link between the central orexin expression and apoptotic pathways related to misfolded protein response and various organelles. Orexin’s anti-inflammatory and autophagy-mediating mechanisms within the central nervous system demonstrate the diverse ways in which orexin responds to stressors within the central nervous system, and facilitates a central response to metabolic states.

In addition to metabolic and stress states, orexins have been implicated in the sleep-wake cycle (Figure 1). Orexin-producing neurons have been shown to project fibers to the brainstem and thalamus, including the locus coeruleus and the raphe nucleus [11,38]. These locations are centers for regulating arousal and suggest that orexins have a role in sleep-wake cycle regulation. In Labrador retrievers and Doberman pinchers, familial canine narcolepsy was revealed to be due to a mutation in OX_2_R [39]. Additionally, i.c.v. injection of both orexins in rats increased wakefulness and decreased sleep [40,41]. A mouse model for childhood narcolepsy, which involved selective death of orexin neurons, revealed that the reason children experience narcolepsy more severely than adults is not due to age at time of orexin neuron loss, but the rapid speed by which these neurons are lost in children compared to adults [42]. This study and others demonstrate the critical nature of orexin-responsive neurons to sleep-wake cycles in mammals. Natural stimuli can also affect the orexin system and its relationship to the sleep-wake cycle. In diurnal Nile grass rats, the levels of orexin-A and orexin immuno-reactive neurons was increased at night and in animals housed in bright light compared to dim. Additionally, bright light environments resulted in an increased protein expression of both orexin receptors in the medial prefrontal cortex in males compared to dim light [43]. These studies demonstrate orexin’s role in sleep-wake cycles in mammals, as well as the orexin response to stimuli, in addition to their influence on feeding behavior.

### 3.2. Central Orexin Signaling Pathways

**Figure 2:**
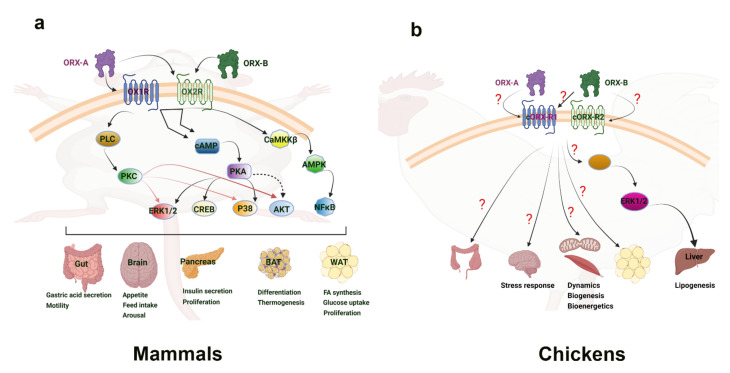
Orexin signaling pathways in mammalian (**a**) and avian species (**b**). “?” means that the downstream mediators are still not known or not well defined. Clear connections have been made to the link between orexin and the activation of energy sensing AMPK within the central nervous system of mammals. This provides a basis for connecting feeding behavior with orexin. Additionally, the ERK1/2 and Akt signaling pathways have been seen in hepatic response to orexin, inducing diverse cellular responses. While the main mechanism of action has yet to be determined, adipose tissue is shown to increase cytokines and other factors in response to orexin. In avian species, the ERK1/2 pathway has also been implicated in the liver response to orexin. Several mitochondrial related genes were shown to respond to orexin in avian muscle, but the direct mechanism of impact has yet to be elucidated. Culpable molecular signaling pathways have yet to be discovered in the avian central nervous system and adipose tissue response to orexin. AKT—Ak strain transforming kinase; AMPK—adenosine monophosphate-activated protein kinase; BAT—brown adipose tissue; CaMKKβ—Calcium/Calmodulin dependent protein kinase; cAMP—cyclic adenosine monophosphate; cORX-R1; chicken orexin receptor 1; cORX-R2—chicken orexin receptor 2; CREB—cAMP-response element binding protein; ERK1/2—extracellular-signal-regulated kinase 1/2; NFκB—nuclear factor kappa light chain enhancer of activated B cells; ORX-A—orexin-A; ORX-B—orexin-B; OXR1—orexin receptor 1; OXR2—orexin receptor 2; PKA—protein kinase A; PKC—protein kinase C; PLC—phospholipase C; P38—mitogen-associated protein kinase 38; WAT—white adipose tissue.

The molecular mechanisms of central orexin’s mode of action have also been investigated. In energy restricted dairy cows, orexin A neurons are colocalized with adenosine monophosphate-activated protein kinase (AMPK) and peroxisome proliferator-activated receptor (PPAR) gamma. Additionally, energy restriction phosphorylates AMPK, leading to AMPK’s activation and to an increased PPARγ expression, indicating that orexin-A’s control of energy homeostasis in dairy cows involves AMPK [44]. AMPK is an enzyme that acts as a sensor for cellular energy status through changes in the ATP-to-AMP ratio and activating downstream targets to induce metabolic shifts [45,46]. AMPK is known to affect fat metabolism and glucose utilization, as well as impact the balance between catabolic and anabolic pathways within the cell [46]. PPARγ is a receptor whose ligands are known as potent insulin sensitizers, and it is known for its involvement in the mobilization of lipids and glucose metabolism [47]. It was later shown that orexin-A activated hypothalamic AMPK signaling in a calcium-dependent manner via a voltage-gated L-type calcium channel (Figure 2) [48]. This research points to orexins’ ability to directly activate AMPK signaling within the central nervous system, providing a means in which feeding behavior and energy homeostasis are linked. A link between central regulation and energy homeostasis was also established via hypothalamic orexin expression and brown adipose tissue thermogenesis. AMPK inhibition in the ventromedial nucleus of the hypothalamus, followed by increased orexin signaling in the lateral hypothalamic area, was seen under thermogenic effects induced by bone morphogenetic protein (BMP) 8B in mice. The thermogenic effect of BMP8B is due to its impact on the browning of white adipose tissue, and both its thermogenic effect and the effects on orexin expression were reduced by the knockout of glutamate vesicular transporter 2 (VGLUT2) [49]. These findings show the central control of energy homeostasis via orexin’s relationship to AMPK within the hypothalamus.

### 3.3. Orexin in the Avian Central Nervous System

The central role of orexins in avian species is a stark contrast to mammalian species. In fact, i.c.v. administration of orexins in neonatal chicks failed to stimulate appetite and food intake [50]. This is despite the fact that orexin-A and -B are highly conserved among vertebrates, as evidenced by the predicted amino-acid sequence of chicken prepro-orexin [51]. Additionally, while orexin-positive cell bodies were found in the periventricular hypothalamic nucleus and extending into the lateral hypothalamic area in chicken, fasting had no effect on the orexin mRNA expression [51]. These phenomena were further investigated upon research into the chicken orexin receptor (cOXR). It was found that cOXR corresponds more closely to OX_2_R in mammals, with approximately 80% similarity [14]. cOXR was found to be widely expressed throughout the bird brain, and particularly abundant in the cerebrum, hypothalamus, and optic tectum [14]. It was later found that orexin-A and -B do not seem to be involved in the wake-sleep cycle, as their expression levels did not change in the brains of sleeping vs. awake laying hens [52]. Within the avian brain, orexin-A and -B neurons have been found central on the paraventricular nucleus and extending into the lateral hypothalamic area in several birds [53,54]. The highest density of orexin neurons in the house finch was found within the preoptic area, the hypothalamus, and the thalamus, with projections also found in the third ventricle caudally [54]. The distribution of orexin neurons in varying regions within the avian brain point to a diverse involvement of orexin in numerous behavioral and centrally-regulated functions. A recent study by Wei and colleagues showed that keel fracture induced stress and inflammation, along with a reduced expression of the orexin system in the hypothalamus of laying hens [55]. This would implicate a central expression of orexin in stress responses, similar to what was seen in mammalian species. On the other hand, Lei et al. reported that acute heat stress did not elicit any change to the hypothalamic expression of orexin mRNA in broiler chickens [56]. Taken together, these studies suggest a potential CNS role for the avian orexin system in certain stress responses, but not in energy balance, and this role may be strain-dependent (layers vs. broilers). Therefore, further in-depth investigations are needed to elucidate the mechanisms in which orexin operates within the avian CNS and its downstream effects.

## 4. Peripheral Role of Orexin

Orexin-A and -B are expressed in numerous mammalian and avian peripheral tissues. The function of orexin and its receptors in these various peripheral tissues has become a new source of investigation and discovery (Figure 1).

### 4.1. Intestinal Tract and Digestion

A major tissue of interest was the intestinal tract, given orexin’s relationship to feeding behavior and energy status. In the intestines of rats, orexins are regulated by nutritional status and affect insulin secretion and intestinal motility [20]. Additionally, in rats, mice, guinea pigs, and humans, mRNA of prepro-orexin, orexin-A, orexin-B, and both receptors, have been found in the endocrine cells of the ileal mucosa [57,58,59]. Furthermore, orexins in the intestinal tract regulate intestinal motility in all these species [20]. Orexin-A induced contraction in dissected rat jejunal and mouse duodenal, jejunal, and ileal tissues [60,61]. Contractions induced by orexin-A were inhibited by an OX_1_R antagonist in mice, indicating the necessity of this receptor in intestine motility control [62]. Indeed, intracellular calcium signaling was induced in isolated rat duodenal enterocytes primarily through OX_1_R, and enterocytes from food deprived rats showed a marked decrease in receptor expression and subsequent calcium signaling from orexin-A [63]. However, some studies showed orexin-A relaxation when administered in the presence of atropine and guanethidine [51]. The contrasting results indicate a complex network of signaling needing to be fully elucidated to better understand the role orexins play in intestinal motility. Orexins within the intestinal tract also influence gastric and duodenal secretions, but through primarily central regulations [19,20].

Other organs where orexin plays a role in digestion and metabolism are the pancreas, adrenal gland, kidney, adipose tissue, liver, and muscle [20,64,65]. There have been conflicting data on the role of orexin in the pancreas related to glucose metabolism. Isolated rat islets showed no response to orexin-A until high doses, which decreased glucose-stimulated insulin secretion [66]. Intravenous infusion of orexin-A in fasted rats significantly increased plasma glucagon, while decreasing plasma insulin levels within 20 min [66]. In contrast, isolated rat islets were shown to respond to high doses of orexin-B treatment with increased insulin secretion when at basal and high glucose concentrations, and low-dose orexin-A stimulated insulin secretion at both glucose levels [67]. The contrasting results suggest that orexins may affect glucose homeostasis via pancreatic hormone secretion, which is dependent on glucose availability and other currently unknown factors, warranting further research.

### 4.2. Adipose Tissue

In addition to the pancreas, orexin has been found to be present and functional within adipose tissue. Both receptors were found in rat adipocytes, and it has been shown that obese rats had reduced OX_1_R protein production within adipocytes compared to non-obese rats [68,69]. Porcine preadipocytes, and subcutaneous and visceral fat also express both orexin receptors at the protein and mRNA level [23]. However, OX_1_R expression was higher than OX_2_R in the fat depots and isolated adipocytes [70]. In porcine preadipocytes, orexin-A and -B enhanced differentiation, as shown by the increased proadipogenic genes and lipid accumulation [23] (Figure 1). However, their role in rat preadipocyte differentiation is less clear, with orexin-A failing to affect preadipocytes but OX_1_R mRNA expression increasing during differentiation [68,71]. Orexins have also been implicated in hormone production by adipose tissue, which can regulate metabolism and the pathophysiology of obesity and insulin sensitivity. Orexin-A was reported to stimulate the expression and secretion of adiponectin in mouse adipocytes [68]. Orexin-A also increased plasma adiponectin levels in lean, obese, and type 2 diabetic animals [72]. In porcine adipocytes, orexin-A stimulated leptin secretion and expression, while in rat and mice cells, leptin levels were reduced [70,73,74]. Key pro-inflammatory cytokines, visfatin, TNFα, and resistin were also significantly reduced in the plasma of orexin A-treated obese and type 2 diabetic rats [72]. These results suggest a modulation of obesity related factors and metabolism by orexin in the adipose tissue.

### 4.3. Liver

Another key organ related to fat metabolism and other physiological processes is the liver. Indeed, the orexin knock-out mice had increased weight gain compared to the wild-type mice, even with comparable feed intake, due to differences in lipid metabolism. The livers of orexin knock-out mice were significantly larger with increased lipid accumulation and hepatic triglyceride content. These physiological differences coincided with increased hepatic lipogenic gene expressions and lower fatty acid transporter genes [75,76]. Additionally, chronic high caloric intake in rats resulted in increased renal OX_1_R protein expression, along with increased nicotinamide adenine dinucleotide phosphate oxidase (NOX) subunit 4 expression in the liver, with hypertension, indicating a role for renal and hepatic orexin and stress response factors [77]. Orexin-A also was shown to induce glucose transporter (GLUT) 4 at the protein and gene level in the liver of the orange-spotted grouper in a glucose-dependent manner, indicating orexin’s involvement in glucose utilization within the livers of fish [78]. These findings suggest a key role of orexin in hepatic fat metabolism and glucose utilization, warranting further research into how orexin functions within the hepatic system and its downstream effects.

### 4.4. Peripheral Orexin Signaling Pathways

As previously described, orexin showed connections to obesity-related pro-inflammatory factors such as visfatin, TNFα, and resistin. These factors are related to pro-inflammatory signaling pathways that have also been implicated in the liver. The stimulation of GLUT4 production in hepatocytes from orange-spotted grouper was blocked by extracellular signal-regulated protein kinase (ERK) 1/2, c-Jun N-terminal kinase (JNK), or p38 mitogen-activated protein kinase (MAPK) inhibitors, indicating the role of these signaling pathways in hepatic glucose utilization in fish [78]. These pathways were also implicated in the avian liver. A recent study by Dridi’s group using predicted OX_1_R for chicken and cOXR (most similar to OX_2_R) showed that orexin is localized within the endoplasmic reticulum, Golgi apparatus, and lysosomes in avian liver cells. Additionally, treatment with recombinant orexins increased fatty acid synthase protein levels in vivo and in vitro, while also activating acetyl-CoA carboxylase, malic enzyme, and ATP citrate lyase [64]. These effects were also attenuated by blocking ERK1/2 activation, indicating orexin’s ability to induce hepatic lipogenesis through ERK1/2 signaling cascades [64] (Figure 2). ERK is a mitogen-activated protein kinase responsible for delivering extracellular signals to the nucleus via translocation, therefore regulating cell cycle, proliferation, and development [79]. ERK and other mitogen-activated protein kinases, such as p38 MAPK, have numerous cellular functions including inflammatory and stress responses, cellular differentiation, and cell death pathways [80]. Within the liver, it is clear that for many species, orexin’s effects on metabolism and glucose utilization are dependent on intracellular signaling pathways, such as ERK1/2, responsible for cellular function, proliferation, and stress response.

Other studies have shown another pathway to be implicated, tied to the ERK1/2 signaling cascade. Orexin knock out mice exhibited impaired insulin induced Akt phosphorylation in the hypothalamus, skeletal muscle, and liver, as well as phosphorylation states of upstream and downstream molecules within the Akt signaling pathway [81]. This indicated how deficiency in orexin contributes to peripheral insulin resistance. The Akt pathway has also been implicated under orexin-A treatment in the granulosa cells of mice, where orexin-A and OX_1_R impacted the phosphorylation of this pathway and had impacts on downstream proliferation and apoptosis [82]. The Akt pathway, while it can be linked to ERK1/2 upstream, resulted in varying and different cellular responses than the ERK1/2 pathway [83]. Further research is needed to elucidate the mechanisms through which orexin targets and acts within tissues, the differences in these pathways could explain the varying effects of orexin that are species- and tissue-dependent.

### 4.5. Orexin in Avian Peripheral Tissues

Research into orexin’s peripheral effects is still limited in avian species, despite it looking more promising than central regulation. In chickens, immunohistochemical localization and distribution of orexin-A and -B was found in the endocrine cells, nerve fibers, and neurons of the stomach and intestines, along with the protein expression of prepro-orexin and both receptors [84]. This study indicates a potentially similar role of orexin in avian intestinal tract secretions and function as is seen in mammals.

In avian hepatoma cells, orexin-A decreased the visfatin expression while orexin-B had no significant effect [85]. Additionally, both oxidative and heat stress were shown to alter the expression of orexin and its receptors in avian species, with oxidative stress appearing to modulate post-transcriptional mechanisms of orexin regulation [86]. These results provide a basis for orexin influencing the peripheral mechanisms of metabolism in avian species, specifically through regulating secretions and functions in the liver.

The muscle was also investigated in the avian species as a main area of selection in modern broilers, leading to high metabolic demands within the muscle. To that end, the orexin system was shown to be expressed in the muscle and to regulate mitochondrial dynamics via fission- and fusion-related genes and their associated transcription factors [22]. In addition to the regulation of the orexin system within avian muscle, orexin treatment was also shown to impact mitochondrial biogenesis and functional genes, while also significantly decreasing the proton leak within a quail muscle cell line [22]. In quail muscle, acute heat stress decreased the expression of orexin and its receptor [87]. In contrast to lack of central effects, these studies showed that orexin exerts peripheral effects on metabolism, energy homeostasis, and inflammatory factors. Further research is needed to elucidate the extent to which the orexin system works within avian physiology.

In addition to physiological implications, the impact of nutrition on the orexin system has limited investigation. A recent study has shown that dietary chromium increased orexin and GLUT expression levels and reduced levels of NF-κB and HSPs in the ovaries of heat-stressed laying hens, indicating nutritional interventions capable of modulating the orexin and inflammatory systems [88]. Further research into the impacts of nutrition on the orexin system within the peripheral tissues of chickens is warranted, and could provide insight into the mechanisms of improved stress response and energy homeostasis.

## 5. Conclusions and Perspectives

The orexin system was first discovered because of its central regulation of feeding behavior and wake-sleep cycles, but has since been shown to have far-reaching effects on metabolism, energy homeostasis, and stress responses. In mammalian species, orexins exert a central control on feed intake and sleep, while also exhibiting peripheral effects on pancreatic secretions and adipocyte differentiation. Within the central nervous system, the AMPK pathway provides a clear link to energy homeostasis and neurological influence, and has been shown to be a key mediator for orexin action in mammals. For peripheral tissues, the liver responds to orexin via ERK1/2 pathways. In avian species, orexin has only recently been investigated, but already demonstrates a diverse and pleiotropic role in muscle energy metabolism and mitochondrial dynamics, hepatic lipogenesis and stress response, and cytokine secretions. However, far less is known regarding orexin’s role in avian gut and adipose tissue (Figure 1 and Figure 2). Nonetheless, orexin research has clearly demonstrated the dynamic nature of central and peripheral crosstalk via neuropeptides such as orexin. The functions and effects of orexin in animals show connections to obesity, glucose utilization, metabolic homeostasis, and behavior. In poultry, the fast growth, insulin resistance, and metabolic demands are linked to obesity, glucose utilization, and metabolic homeostasis, of which orexin has clear functions in. Therefore, future research into the mechanisms through which orexin exerts its effects is warranted, particularly in minimally researched avian species, as a means of utilizing the orexin system to improve animal breeding, husbandry, nutrition, and health.

## Data Availability

No new data was created during this study.
